# Acute myelogenous leukemia switch lineage upon relapse to acute lymphoblastic leukemia: a case report

**DOI:** 10.1186/1757-1626-2-154

**Published:** 2009-10-15

**Authors:** Elisa Dorantes-Acosta, Farina Arreguin-Gonzalez, Carlos A Rodriguez-Osorio, Stanislaw Sadowinski, Rosana Pelayo, Aurora Medina-Sanson

**Affiliations:** 1 Department of Pediatric Hematology and Oncology, Hospital Infantil de Mexico Federico Gomez, Mexico City, Mexico; 2 Department of Critical Care Medicine, Instituto Nacional de Ciencias Medicas y Nutricion Salvador Zubiran, Mexico City, Mexico; 3 Department of Pathology, Hospital Infantil de Mexico Federico Gomez, Mexico City, Mexico; 4 Oncology Research Unit, Oncology Hospital, National Medical Center, IMSS, Mexico City, Mexico

## Abstract

Acute leukemia, the most common form of cancer in children, accounts for approximately 30% of all childhood malignancies, with acute lymphoblastic leukemia being five times more frequent than acute myeloid leukemia. Lineage switch is the term that has been used to describe the phenomenon of acute leukemias that meet the standard French-American-British system criteria for a particular lineage (either lymphoid or myeloid) upon initial diagnosis, but meet the criteria for the opposite lineage at relapse. Many reports have documented conversions of acute lymphoblastic leukemia to acute myeloid leukemia.

Here, we report the case of a 4-year-old child with acute myeloid leukemia, which upon relapse switched to acute lymphoblastic leukemia. The morphologic, phenotypic, and molecular features suggest the origin of a new leukemic clone.

## Introduction

Leukemia is a group of malignant diseases of the hematopoietic system characterized by the uncontrolled overproduction of either immature (acute leukemia) or terminally differentiated (chronic leukemia) leukocytes.

Acute leukemia, the most common form of cancer in children, accounts for approximately 30% of all childhood malignancies [1]. A frequency of ≥ 20% blasts is required to confirm a diagnosis of acute leukemia. The first comprehensive morphologic classification system for acute leukemias was the French-American-British (FAB) classification, which was established in 1976 and revised in 1985. Other recommended tests are immunophenotyping, cytogenetics, and molecular genetics tests [2].

Acute leukemias can be further sub classified by determining whether the malignant leukocytes are of myeloid origin (cells of granulocyte, monocyte, erythroid, or megakaryocyte lineage) or lymphoid origin (B-cells, T-cells) [3].

Overall survival for patients with acute lymphoblastic leukemia is approximately 80%; currently for acute myeloid leukemia (AML), most large studies show a five-year event-free-survival rate of almost 50%, despite the fact that between one third and one half of patients with AML experience relapse, and no standard therapy is recognized for patients with relapsed and/or refractory AML. Relapses mostly exhibit the same FAB subtype. Patients with relapsed/refractory AML usually respond less well and for a shorter duration to reinduction therapies. This clearly points to the induction of drug-resistant mechanisms and is, in part, related to specific chromosomal abnormalities and the duration of the first remission [4,5].

However, unexplained variability in clinical course still exists among some individuals within defined risk-group strata.

"Lineage switch" is the term that has been used to describe the phenomenon of acute leukemias that meet standard FAB criteria for a lineage (lymphoid or myeloid) at initial diagnosis but upon relapse meet the criteria for the opposite lineage [6].

Most reports of lineage switching have demonstrated acute lymphoblastic leukemia (ALL) to acute myeloid leukemia (AML) conversions [7-9]. The prognosis for these patients is variable, and there is no standard treatment for them.

## Case presentation

A 4-year-old Mexican Mestizo (Hispanic) male was admitted to the Hospital Infantil de México Federico Gómez presenting a 30-day history of fever, pallor, and enlarged cervical lymph nodes.

The initial laboratory findings revealed a white blood cell count of 4,300/mm^3^, with 16% blasts in the peripheral blood, 8.8 g/dL hemoglobin, and a platelet count of 49,000/mm^3^.

**Figure 1:**
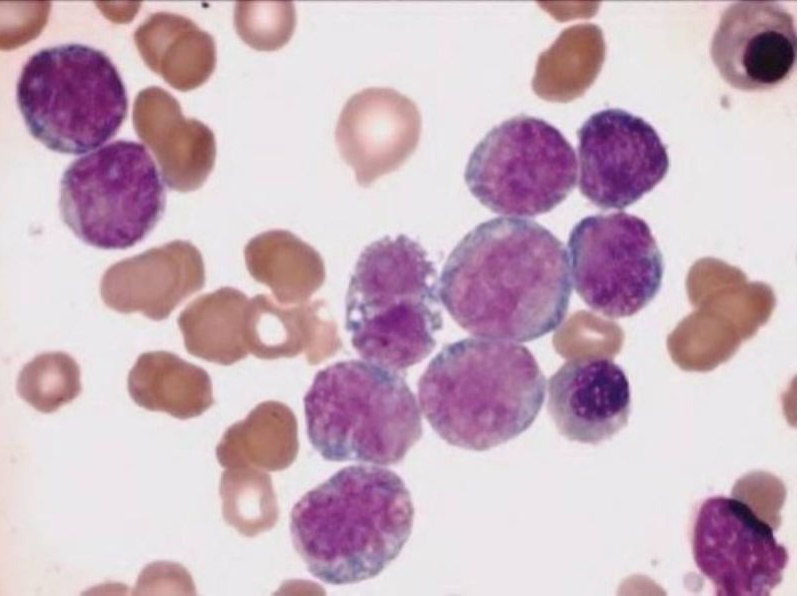
**Bone marrow aspiration at AML diagnosis**. Blast cells were large in size and had a low nucleus:cytoplasm ratio, monocytoid aspect, and blue-gray cytoplasm. Wright stain, 1000×.

**Figure 2:**
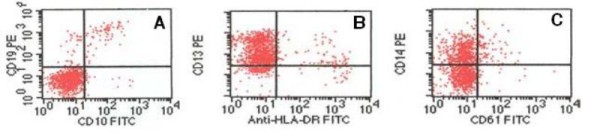
**Flow cytometry AML at diagnosis**. Immunophenotyping of leukemic cells by flow cytometric analyses revealed myeloid blasts negative for CD10 and CD19 **(A) **but positive for CD13 **(B) **and CD14 **(C)**.

Cerebrospinal fluid was negative for blast cells. The bone marrow was hypercellular, with 64% blasts, which were large in size and showed low nucleus: cytoplasm ratio, monocytoid features, and blue-gray cytoplasm; > 80% of the blast cells were monoblasts, and the rest were promonocytes or monocytes (Figure 1). The surface immunophenotype was CD45^+ ^(91.1%), CD15^+ ^(95.4%), CD14^-/+ ^(37.7%), CD13^+/- ^(78.5%), HLA-DR^+/- ^(78.5%), CD22^-/+ ^(42.8%), CD10^- ^(2.1%) CD19^- ^(2.5%) CD20^- ^(0.7%) CD7^- ^(0.07%), CD3^-/+ ^(19.4%), and CD33^- ^(7.9%) (Figure 2).

Cytochemistry was positive for myeloperoxidase (MPO). Conventional cytogenetic analysis using direct and 24-hour unstimulated and unsynchronized cultures revealed a 46 XY karyotype. A diagnosis of M5 AML was made. The patient started chemotherapy with a modified MRC-10 protocol: daunorubicin 50 mg/m^2 ^(days 1, 3 & 5); cytarabine 100 mg/m^2 ^in a 1-hour infusion every 12 hours through days 1 to 10; and etoposide 100 mg/m^2 ^in a 4-hour infusion every 24 hours through days 1 to 5.

Due to oral candidiasis and an episode of sepsis secondary to *Acinetobacter iwofii*, the patient was treated with meropenem, amphotericin, and fluconazole.

Remission was determined by marrow aspiration with no blast cells at day 27^th ^after first chemotherapy cycle.

Thirty days after the first course of chemotherapy, a second chemotherapy cycle was administered without complications daunorubicin 50 mg/m^2 ^days 1, 3 & 5; cytarabine 100 mg/m^2 ^in a 1-hour infusion every 12 hours through days 1 to 4; and etoposide 100 mg/m^2 ^in a 4-hour infusion every 24 hours through days 1 to 5.

A third cycle: etoposide 100 mg/m^2 ^in a 4-hour infusion every 24 hours on days 1-5 and cytarabine 1000 mg/m^2 ^in a 1-hour infusion every 12 hours (days 1-3) was stopped on day 3 because of a life-threatening episode of pneumonia; the patient was transferred to the ICU and supported by mechanical ventilation. Because a lung CT scan showed features of a right lung abscess, a medium and right lobectomy was performed.

Upon clinical and hematologic recovery, the patient received a fourth cycle of chemotherapy with mitoxantrone 10 mg/m^2 ^in a 1-hour infusion every 24 hours (days 1-5) and cytarabine 1000 mg/m^2 ^in a 1-hour infusion every 12 hours (days 1-3). Two weeks later, an episode of fever, along with neutropenia and mucositis, was recorded, and the patient was treated with cefepime and amikacin.

The patient received a fifth cycle or chemotherapy: etoposide 100 mg/m^2 ^in a 4-hour infusion every 24 hours for 5 days and cytarabine 1000 mg/m^2 ^in a 1-hour infusion every 12 hours for 3 days.

Seven months later, after the diagnosis, the chemotherapy protocol was completed with a clearance of blast cells.

**Figure 3:**
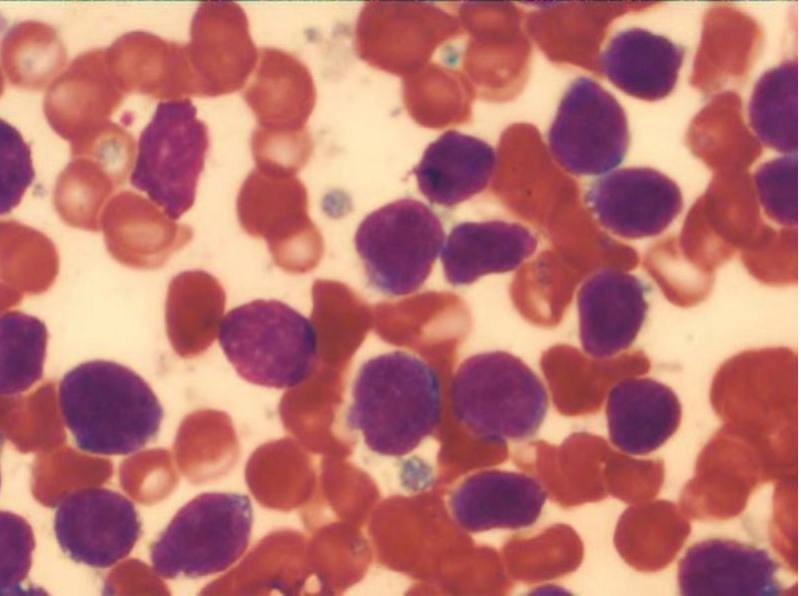
**Bone marrow aspiration at relapse**. Blasts were smaller, with a predominance of small cells, scanty cytoplasm, moderate cytoplasmic basophilia, and variable cytoplasmic vacuolation. Wright stain, 1000×.

**Figure 4:**
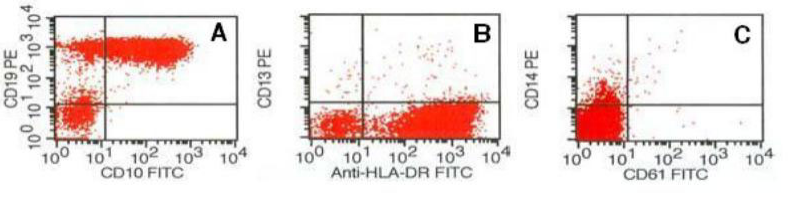
**Flow cytometry at relapse**. Flow cytometric analyses of leukemic cells at relapse. Blast cells were identified as lymphoblasts because the immunophenotype was positive for CD10 and CD19 **(A) **but negative for CD13 and CD14 **(B, C)**.

Two months later, the patient presented at the hospital with petechiae. Initial laboratory data revealed a white blood cell count of 7,700/mm^3^, 13% blast cells, 13.7 g/dL hemoglobin, and a platelet count of 33,000/mm^3^. The marrow was hypercellular and contained 85% blasts. In contrast to the previous neoplasm, the blasts in the current specimen were smaller, with a predominance of small cells, scanty cytoplasm, moderate cytoplasmic basophilia, and variable cytoplasmic vacuolation (Figure 3), along with the immunophenotype CD45^+ ^(81.6%), CD10^+ ^(77%), CD19^+ ^(89%), CD22^- ^(0%), CD20^- ^(3.7%), CD15^- ^(0%), CD14^- ^(4.5%), CD7^- ^(7.8%), CD3^- ^(13.1%), CD13^- ^(0.27%), CD33^- ^(0.62%), and HLA-DR^- ^(0.2%) (Figure 4). These lymphoblasts were negative for myeloperoxidase (data not shown).

The patient was diagnosed with pro-B lymphoblastic leukemia. Cytogenetic studies performed on the marrow aspirate revealed a karyotype of 46 XY.

We tried to induce remission with the national protocol for acute lymphoblastic leukemias, adapted from TOTXV protocol from St Jude Children's Research Hospital with vincristine 2 mg/m^2 ^once a week for four weeks, daunorubicin 30 mg/m^2 ^once a week for two weeks, L-asparaginase 10,000 IU/m^2 ^three times a week for a total of nine doses, and dexamethasone 6 mg/m^2 ^for four weeks. However, the bone marrow examination on the 14^th ^and 21^th ^days of induction showed persisting infiltration with 50% blasts, and the patient remained pancytopenic. After an episode of fever, abdominal pain, and diarrhea, antibiotic therapy was conducted.

As the bone marrow examination showed persisting infiltration, we indicated cycle 1 and 2 to myeloid leukemia (described above). This scheme of chemotherapy is used in our hospital for patients with early relapse or refractory acute leukemia. After that, the patient had infectious complications, followed by development of tumor lysis syndrome and septic shock. Chemotherapy was stopped and the patient was transferred to the ICU and supported by mechanical ventilation. After clinical recovery, the bone marrow showed persisting infiltration with 85% blasts, and the patient's parents asked for palliative care; the child was cared for at home for four weeks under the surveillance of a palliative care clinic. Despite supportive measures, he died six months after the diagnosis of relapse. No autopsy was authorized by the family.

**Figure 5:**
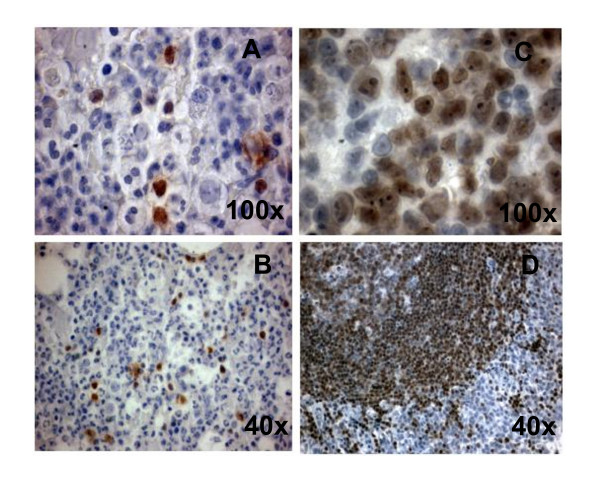
**PAX5 expression in bone marrow from pro-B ALL at AML remission**. Immunohistochemistry for PAX5 was performed on a marrow biopsy. The frequency of PAX5 cells was 15% **(A, B)**. PAX5 expression by lymph node germinal center cells is shown for control **(C, D)**.

In order to investigate if a B lymphoid transcription factor was already expressed at the time of remission of the myeloid leukemia, we performed detection of PAX5 (B-cell-specific activator protein for B cells, including B-lymphoblastic neoplasms) [10], in bone marrow biopsy taken at the end of the first scheme of chemotherapy, when the patient started surveillance (Figure 5).

Briefly, one marrow biopsies fixed in Bouin's solution were decalcified, and paraffin-embedded tissue sections were stained with an antibody against the PAX5. The immunocytochemistry revealed scanty PAX5 expression. (Only 15% of cells in the specimen were positive). The same test was not conducted at relapse because no autopsy was performed.

## Discussion

Lineage commitment and differentiation to a mature cell type are considered to be unidirectional and irreversible processes under physiological conditions. However, a number of studies with murine models suggest that plasticity is a significant attribute of hematopoietic progenitors, and the process of cell formation is much more dynamic than previously thought [11]. Lineage switching is an example of the lineage heterogeneity that exists in some acute leukemias [12]. The frequency of lineage switching among patients with acute leukemia that relapse is estimated to be between 6% and 9% [6].

Some conditions could explain the lineage change at relapse, being one of them the possibility of a second neoplasm, following two or three years of high dose etoposide treatment[13]. Our patient received 1,700 mg/m^2 ^of etoposide in the first chemotherapy cycle, and the relapse occurred only 2 months after treatment. It is less probable to develop a secondary leukemia before a latency period of two years. Another hypothesis to explain immunophenotype changes at relapse is clonal selection. This mechanism has been proposed in patients with a persistent TEL-AML1+ preleukemic/leukemic clone at relapse [14].

We performed karyotype analysis for our patient and did not find any cytogenetic alteration. In some instances, an acute leukemia lineage switch may represent the emergence of a new leukemic clone characterized by different morphology than the observed upon initial diagnosis, without gene rearrangements upon conversion to another lineage. Zardo et al. propose that the plasticity and reversibility of modifications affecting chromatin structure are important in the expression of genes involved in cell fate decisions and in maintenance of cell-differentiated states. Epigenetic changes in DNA and chromatin, which must occur to allow accessibility to transcription factors at specific DNA-binding sites, are regarded as emerging major players for hematopoietic stem cell lineage differentiation [15].

Mantadakis et al. [16] described the in vivo conversion of T-cell acute lymphoblastic leukemia (ALL), with an early thymocyte immunophenotype and no myeloid markers, to acute myeloid leukemia (AML). This evidence suggests that a subset of T-cell leukemias with minimal differentiation can display a relapse as another lineage, AML.

Palomero et al. suggest that the leukemic transformation may occur in early progenitors and might be influenced by external and internal clues; they propose mutations in the NOTCH1 transcription factor as responsible in lineage switch leukemias [17].

In our patient, lineage markers revealed the emergence of apparently new lymphoid populations. In this case the new 'relapse lymphoid clone' was not detected at the moment of surveillance in between the first and secondary diagnosis by investigation of the B related transcription factor PAX5. The selection and emergence of chemo-resistant sub-clones that are undetectable by routine methods, or selection of a new leukemic clone have been previously reported by Henderson et al [18], and we can not ruled out either of this possibilities for our patient.

Studies of normal blood cell development and malignant transformation of hematopoietic cells have shown that the correctly regulated expression of stage- and lineage-specific genes is a key issue in hematopoiesis. The transcription factor PAX5 has been shown to be a B-cell-specific activator protein and seems to be crucial for B lymphopoiesis, including B-lymphoblastic neoplasms [19,20].

The immunocytochemistry for PAX5 suggests that at least at the moment of the clinical remission, there was no expression of a transcription factor of lymphoid origin, and between the first and second leukemias, there was a period of time where there were no data supporting lymphoid malignancy until the patient relapsed.

The absence of a lymphoid transcription factor at the beginning of surveillance suggests that the lineage switch occurred upon relapse. These data put forward the possibility of *de novo *lymphoid leukemia after myeloid leukemia. Further studies on the mechanisms of lineage commitment and differentiation in acute leukemia cases will help us to understand the cell and molecular biology of this phenomenon.

## Conclusion

Here, we present one case of lineage switch, from AML to ALL which we consider of great interest due to the importance of early recognition of the specific leukemic lineage, and the medical aid necessary for appropriate treatment. Lineage switching is a rare but well-documented phenomenon. The change in morphology, the cell phenotype at relapse, and the absence of a crucial lymphoid transcription factor when the patient was under clinical surveillance all suggest that a lineage switch from AML to ALL might represent a heterogeneous original clone at the molecular level or the emergence of a second new leukemic clone that lacks molecular heterogeneity.

## Competing interests

The authors declare that they have no competing interests.

## Authors' contributions

EDA analyzed and interpreted the patient data regarding the hematological disease and the relapse. SS performed the histological examination of the bone marrow. EDA, FAG, CARO, RP, and AMS were major contributors in writing the manuscript and analyzing the results. All authors read and approved the final manuscript.

## Consent

Written informed consent was obtained from the patient's father for publication of this case report and accompanying images. A copy of the written consent is available for review by the Editor-in-Chief of this journal.
